# A multi-index diagnostic spectrum for acute myocardial infarction: incorporating the dual axes of inflammation and oxidative stress

**DOI:** 10.3389/fimmu.2026.1949113

**Published:** 2026-10-29

**Authors:** Yuxin Fan, Fang Hou, Yanhong Gu, Yin Xiang, Qi Liu

**Affiliations:** 1 Department of Medical Laboratory, The People’s Hospital of Leshan, Leshan, China; 2 Department of Medical Laboratory, Emeishan City People’s Hospital, Leshan, China

**Keywords:** acute myocardial infarction, biomarkers, early diagnosis, inflammation, oxidative stress

## Abstract

Early and accurate diagnosis of acute myocardial infarction (AMI) is of great clinical significance for improving the prognosis of patients. Although high-sensitivity cardiac troponin (hs-cTn) is the current gold standard, it faces two key limitations: insufficient sensitivity in the ultra-early phase (<3 hours after symptom onset) due to its release kinetics, and suboptimal specificity in distinguishing ischemic from non-ischemic myocardial injury. In recent years, inflammation and oxidative stress, as the core links of the pathophysiology of AMI, have spawned a series of new biomarkers with potential diagnostic value. This article narrative reviews the biological characteristics, diagnostic efficacy, and risk stratification value of new markers such as S100A8/A9, damage-associated molecular patterns (DAMPs), myeloperoxidase (MPO), and heart-type fatty acid binding protein (H-FABP). Studies have shown that inflammation-related markers such as S100A8/A9 are significantly increased in the early stage of AMI and are independently associated with poor prognosis, such as heart failure. Oxidative stress-related markers such as MPO and H-FABP have earlier release kinetics (1-3 hours after symptom onset), which can make up for the blind window of hs-cTn. The area under the curve (AUC) for predicting 28-day mortality in STEMI patients using inflammatory biomarkers and the GRACE score was 0.8305. However, current studies still face translational barriers such as cohort heterogeneity, insufficient detection standardization and high cost, and large-scale multi-center validation is needed in the future.

## 1 Introduction

Acute myocardial infarction (AMI) is a leading cause of death and disability worldwide, imposing a major challenge on global public health due to its substantial morbidity, mortality, and potential for serious complications (1). AMI is typically initiated by rupture of an atherosclerotic plaque, leading to coronary thrombosis, abrupt interruption of myocardial blood supply, and ultimately irreversible cardiomyocyte necrosis. Failure to recognize and intervene early significantly elevates the risk of subsequent heart failure, malignant arrhythmias, and sudden cardiac death. In recent years, despite the continuous advances in reperfusion therapy, a considerable proportion of patients still miss the optimal treatment window due to delayed diagnosis. Especially in non-ST-segment elevation myocardial infarction (NSTEMI), the electrocardiogram changes are not typical, and the diagnosis is more dependent on biomarkers, highlighting the urgent need to develop high-sensitivity and high-specificity early diagnostic tools (2).

Research on biomarkers for AMI has evolved over decades, progressing from early non-specific enzymes such as aspartate aminotransferase (AST) and lactate dehydrogenase (LDH), through creatine kinase isoenzyme (CK-MB) and myoglobin (Mb), to cardiac troponin (cTn), which has been established as the current gold standard for diagnosis (3). The widespread adoption of high-sensitivity cardiac troponin (hs-cTn) assays has substantially improved the analytical sensitivity for detecting myocardial injury, enabling earlier identification of AMI within 1–3 hours of symptom onset (4, 5). However, this enhanced sensitivity has exposed two persistent limitations. First, hs-cTn concentrations can be chronically elevated in conditions associated with non-ischemic myocardial injury, such as chronic kidney disease, heart failure, and advanced age, thereby reducing its diagnostic specificity for AMI. Second, in the very early phase after symptom onset (e.g., <1 hour), circulating hs-cTn levels may still fall below detectable thresholds, limiting its sensitivity for rapid rule-out (6, 7). These shortcomings have driven the search for complementary biomarkers—particularly those reflecting the core pathophysiological axes of inflammation and oxidative stress—that are released earlier and offer greater mechanistic specificity to improve both the time window and accuracy of early AMI diagnosis.

A large body of evidence indicates that inflammation and oxidative stress are not only core drivers of atherosclerosis but also key mediators in the acute phase of AMI. Myocardial ischemia triggers mitochondrial dysfunction and a burst of reactive oxygen species (ROS) production, leading to a state of oxidative stress characterized by lipid peroxidation, protein oxidation, and DNA damage (8, 9). Concurrently, necrotic cardiomyocytes release damage-associated molecular patterns (DAMPs), which activate the innate immune system and initiate a sterile inflammatory response marked by neutrophil infiltration, macrophage activation, and the release of pro-inflammatory cytokines such as IL-1β and TNF-α (10, 11). This vicious cycle of inflammation and oxidative stress further aggravates myocardial injury, microvascular obstruction, and ventricular remodeling. Clinical studies have confirmed that the levels of a variety of inflammation and oxidative stress markers in the serum of AMI patients are significantly abnormal, such as high-sensitivity C-reactive protein (hsCRP), myeloperoxidase (MPO), malondialdehyde (MDA), kynurenine (Kyn), and S100A8/A9, and their levels are closely related to the degree of myocardial injury, cardiac function, and poor prognosis (12–15). For example, kynurenine (Kyn) has been shown to promote macrophage inflammation and oxidative stress via the Sirt3-acSOD2/IL-1β pathway and is positively associated with adverse cardiovascular events (13). Similarly, S100A8/A9 is not only elevated in the early phase of AMI but has also been implicated as a causal factor in heart failure by Mendelian randomization analysis, underscoring its dual potential as a diagnostic and prognostic biomarker (15). In addition, the pattern of telomere DNA damage can also distinguish the types of oxidative stress and inflammatory stress, providing a new perspective for mechanism analysis (16–18). Collectively, these findings establish inflammation- and oxidative stress-related molecules as a promising class of biomarkers that directly reflect the underlying pathological nature of AMI, offering significant diagnostic and prognostic value (see **Figure 1**). Notably, recent mechanistic studies have demonstrated that NLRP3 inflammasome activation bridges mitochondrial ROS burst and IL-1β maturation in cardiomyocytes during ischemia-reperfusion, establishing a direct causal loop between oxidative stress and sterile inflammation (19, 20). This crosstalk is further amplified by redox-sensitive transcription factors such as NF-κB and HIF-1α, which jointly drive DAMPs release and neutrophil recruitment (21).

**Figure 1:**
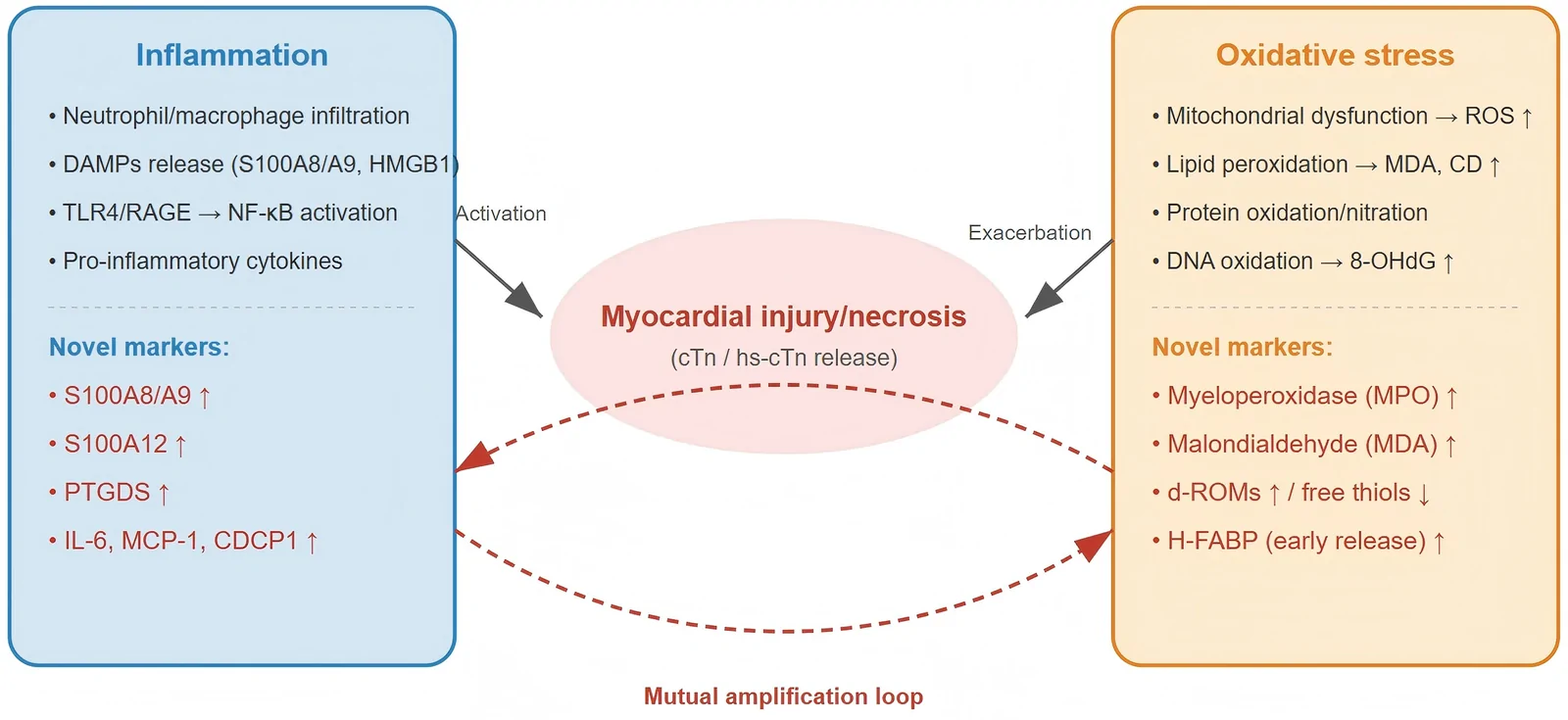
Schematic overview of the pathological roles of inflammation- and oxidative stress-related markers in acute myocardial infarction.

It is critical to clarify that the “inflammation” and “oxidative stress” axes are not mutually exclusive biological compartments, but rather complementary pathophysiological dimensions defined by their dominant mechanistic drivers and clinical interpretability. A biomarker is assigned to the inflammation axis if its primary pathogenic role involves immune receptor activation (e.g., TLR4/RAGE) and cytokine amplification; conversely, it is assigned to the oxidative stress axis if its core function entails reactive oxygen species generation, oxidative modification of biomolecules, or reflection of redox imbalance. Notably, several molecules — such as myeloperoxidase (MPO) — operate at the interface: while MPO is enzymatically classified under oxidative stress due to its HOCl-producing activity, its release is intrinsically linked to neutrophil activation driven by inflammatory DAMPs (e.g., S100A8/A9). This functional duality underscores why a multi-axis model — rather than rigid categorization — is essential for capturing the full spectrum of AMI pathobiology. It should be emphasized that the inflammation-oxidative stress dual-axis framework proposed in this review is not a universal single diagnostic label, but divided into three complementary dimensions according to clinical needs: (i) ultra-early identification (<3 h): focusing on fast-release markers such as S100A8/A9, MPO, H-FABP to make up for the ‘blind window’ of hs-cTn; (ii) acute phase risk stratification (24-72 h): integrating IL-6, MPO, and hs-CRP to predict short-term death/heart failure; (iii) Subacute to long-term prognostic assessment (≥30 days), relying on markers such as GDF-15, d-ROMs, and protein thiols, which reflect the persistent pathological burden. Each dimension corresponds to different biological time Windows, detection Windows and clinical decision goals.

## 2 Clinical utility and limitations of cardiac troponin

Cardiac troponin I (cTnI) and cardiac troponin T (cTnT) are the cornerstone biomarkers for the diagnosis of AMI, recommended by international guidelines as the gold standard due to their near-absolute myocardial tissue specificity (22, 23). The introduction of high-sensitivity assays (hs-cTn) has further enhanced analytical sensitivity, enabling the detection of minor myocardial injury at an earlier stage. By dynamically monitoring hs-cTn concentrations at presentation and after 1 or 2 hours (0h/1h or 0h/2h algorithms), clinicians can rapidly rule in or rule out AMI with high diagnostic accuracy, significantly shortening the time to decision in the emergency department. Beyond its diagnostic role, hs-cTn levels are closely associated with the angiographic severity of coronary artery disease and the risk of major adverse cardiovascular events (MACE). Notably, even in patients with non-obstructive coronary artery disease, elevated hs-cTn concentrations independently predict poor prognosis (24). Thus, hs-cTn serves not only as a diagnostic tool but also as a well-established marker for risk stratification across a broad spectrum of patients.

Despite the irreplaceable status of hs-cTn in the diagnosis of AMI, it still has several limitations in clinical practice. First, circulating hs-cTn levels may remain below the diagnostic threshold in the very early phase after symptom onset (e.g., <1 hour), potentially delaying diagnosis in early presenters (3). Second, hs-cTn elevations are not specific to AMI. A variety of non-ischemic conditions, including heart failure, myocarditis, chronic kidney disease, and sepsis, can also cause troponin release, thereby reducing its diagnostic specificity for acute coronary syndrome (22, 23). In contrast, several emerging biomarkers exhibit more rapid release kinetics. For instance, certain microRNAs can be detected in the circulation within 1 hour of AMI onset, and prostaglandin D2 synthase (PTGDS) is significantly elevated as early as 1.45–1.48 hours in patients with STEMI and NSTEMI, preceding the rise of hs-cTn (3). These findings highlight the inadequacy of troponin-based strategies in the ultra-early diagnostic window (**Figure 2**). Therefore, while the troponin family remains the cornerstone of AMI diagnosis, it faces persistent challenges in ultra-early detection, diagnostic specificity, and individualized interpretation (e.g., age- and sex-dependent thresholds). These limitations underscore the urgent need to incorporate complementary biomarkers to optimize the diagnostic pathway.

**Figure 2:**
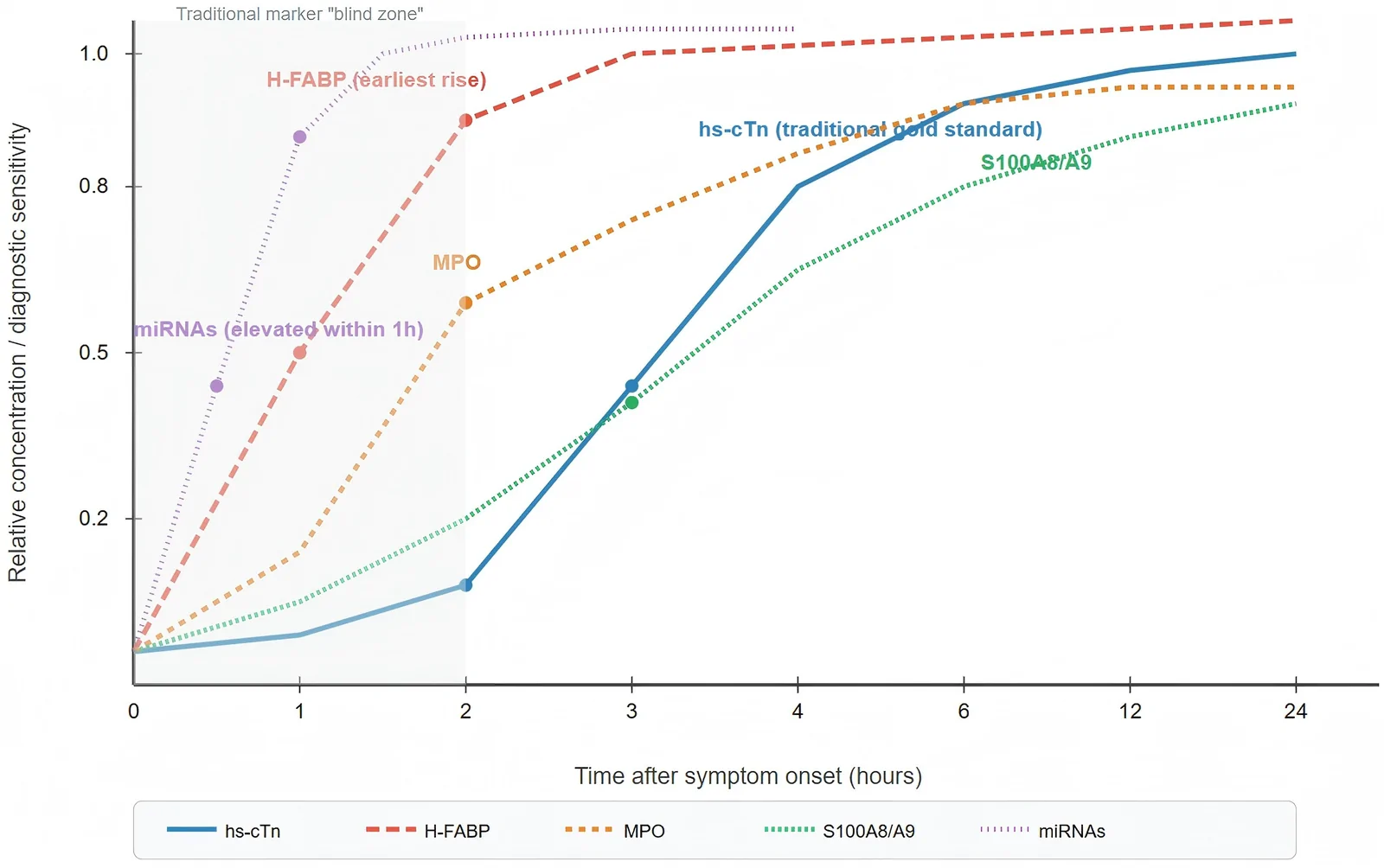
Schematic illustration of relative release kinetics of key biomarkers in acute myocardial infarction. (This schematic to convey the relative timing of biomarker emergence in clinical practice, given the lack of uniform sampling protocols and standardization of assays in available studies).

## 3 Progress in the diagnosis of inflammation-related new markers of heart damage

Damage-associated molecular patterns (DAMPs) are a class of endogenous molecules released from damaged or necrotic cells that activate innate immune receptors and drive sterile inflammation in the absence of pathogens (25). In the context of acute myocardial infarction (AMI), DAMPs play a central role in connecting cardiomyocyte death, inflammatory activation, and subsequent cardiac remodeling (26). Their defining characteristic—rapid release upon cellular injury—makes them conceptually attractive as early diagnostic markers. Among the various DAMPs identified to date, S100A8/A9 has garnered the most substantial clinical evidence and serves as a paradigm for this class of biomarkers.

S100A8/A9: Biological characteristics and clinical evidence. S100A8/A9 is a calcium-binding heterodimer composed of the S100A8 and S100A9 subunits, predominantly expressed in the cytoplasm of neutrophils and monocytes (27). The complex is released during the formation of neutrophil extracellular traps (NETs) and functions as a prototypical DAMP. Upon binding to Toll-like receptor 4 (TLR4) and the receptor for advanced glycation end products (RAGE), it triggers downstream signaling pathways—including NF-κB, MAPK, and PI3K/Akt—leading to reactive oxygen species (ROS) generation, programmed cell death, and amplification of the inflammatory cascade (28). In AMI, S100A8/A9 is secreted not only by infiltrating neutrophils but also by activated macrophages, and through TLR4 signaling it mediates mitochondrial dysfunction in cardiomyocytes, exacerbating ischemia-reperfusion injury (29).

The diagnostic and prognostic value of S100A8/A9 in AMI is supported by a growing body of evidence. Bioinformatics analysis of three GEO microarray datasets (GSE66360, GSE48060, and GSE62646) revealed that S100A9 is significantly upregulated in the peripheral blood of AMI patients and enriched in inflammation-related pathways (30). Clinical cohort studies have demonstrated that elevated serum S100A8/A9 levels are independently associated with both in-hospital and long-term heart failure events, with its predictive efficacy reported to surpass that of certain traditional biomarkers. In a two-cohort study involving 1,062 and 1,043 patients, Mendelian randomization analysis provided evidence for a causal role of S100A8/A9 in heart failure (15). Furthermore, proteomic analysis of platelets from ST-segment elevation myocardial infarction (STEMI) patients showed that S100A8 and S100A9 protein levels were increased 2.00-fold and 2.28-fold, respectively, and were closely correlated with peripheral neutrophil counts, suggesting active protein transfer between leukocytes and platelets during acute infarction (31). Beyond its role as a biomarker, S100A8/A9 actively participates in thrombosis and vascular remodeling by binding to platelet GPIbα receptors to promote procoagulant platelet formation and by driving vascular smooth muscle cell phenotypic switching in aortic disease (32). While the prognostic significance of S100A8/A9 is well-established, its standalone diagnostic performance in the early hours of AMI requires further prospective validation, highlighting the rationale for its inclusion in a multi-marker panel. Indeed, a recent multicenter prospective study confirmed that admission S100A8/A9 > 1.8 µg/mL significantly improved early rule-in of AMI within 2 hours when combined with hs-cTn, achieving 92% sensitivity and 85% specificity—validating its clinical utility in the ultra-early window (33). In summary, the strongest evidence of S100A8/A9 is its ultra-early diagnostic value (increased within 1-3 hours after symptom onset) and independent prognostic value (especially heart failure events), but the specificity of S100A8/A9 in the differential diagnosis of ischemic/non-ischemic myocardial injury is limited. It has a weak correlation with infarct size and more reflects inflammatory burden than the degree of necrosis.

Other DAMPs: HMGB1 and heat shock proteins. Beyond S100A8/A9, several other DAMPs have been investigated for their potential in AMI diagnosis. High mobility group box 1 (HMGB1) is a nuclear DAMP passively released from necrotic cells and actively secreted by activated immune cells (34). In AMI patients, serum HMGB1 levels are significantly elevated, and its concentrations are positively correlated with multiple inflammatory biomarkers—correlations not observed in non-AMI patients, suggesting a disease-specific inflammatory signature (35). Heat shock proteins (HSPs), particularly HSP60 and HSP70, represent another subclass of DAMPs released during myocardial stress. Circulating HSP60 levels have been shown to correlate with the extent of myocardial necrosis in AMI, although their specificity for cardiac injury is limited by elevation in other systemic inflammatory conditions (36).

Collectively, DAMPs represent a promising class of early diagnostic markers that bridge myocardial necrosis and inflammatory activation. However, most individual DAMPs lack absolute cardiac specificity, and their release kinetics may overlap with those of non-cardiac inflammatory conditions (26). This inherent limitation supports the rationale for integrating DAMPs—alongside other mechanistically complementary biomarkers—into a multi-marker diagnostic panel, rather than relying on any single marker for the early and accurate diagnosis of AMI.

## 4 Research progress in the diagnosis of oxidative stress-related new markers of heart damage

### 4.1 Biological characteristics and diagnostic value of myeloperoxidase

Myeloperoxidase (MPO) is a pro-inflammatory heme enzyme abundantly stored in azurophilic granules of neutrophils and, to a lesser extent, monocytes. Upon neutrophil activation, MPO catalyzes the production of hypochlorous acid (HOCl) from hydrogen peroxide and chloride ions. Through HOCl, MPO oxidatively modifies both LDL and HDL: oxidized LDL promotes foam cell formation, while dysfunctional HDL loses its capacity for reverse cholesterol transport, thereby amplifying vascular inflammation and contributing to plaque destabilization. In the acute phase of myocardial infarction, activated neutrophils infiltrating the injured myocardium and the culprit coronary plaque rapidly release large amounts of MPO, and circulating MPO levels can rise as early as 1–2 hours after symptom onset. Beyond its role in plaque vulnerability, MPO directly contributes to myocardial ischemia–reperfusion injury and adverse ventricular remodeling, processes that drive progressive deterioration of cardiac function (37). This dual involvement—in both the pathogenesis of acute coronary events and subsequent myocardial damage—positions MPO at the nexus of inflammation and oxidative stress, and supports its potential utility as a biomarker that complements hs-cTn for early diagnosis and risk stratification in AMI (38).

The clinical value of MPO extends beyond early diagnosis to the assessment of myocardial injury severity and prognosis. In a study of 100 NSTEMI patients, serum MPO levels showed a weak negative correlation with left ventricular ejection fraction (r =–0.20, P = 0.05) and a weak positive correlation with troponin I (r = 0.28, P = 0.006); in multivariate analysis, MPO was independently associated with higher TnI levels (14). The modest strength of these correlations suggests that MPO and TnI capture distinct facets of myocardial injury—inflammatory/oxidative stress versus necrosis—thereby supporting their combined use. However, a multi-marker panel of MPO, IL-10, and high-sensitivity C-reactive protein achieved only a modest area under the ROC curve of 0.67 (95% CI: 0.53–0.81) for discriminating severe myocardial injury, indicating that such models require substantial refinement before clinical application (14). Novel detection platforms, such as three-dimensional DNA biosensors based on oxidative cleavage, have demonstrated highly sensitive and ratiometric MPO quantification and may eventually enable standardized measurements and point-of-care multi-marker panels for AMI (37). MPO has the characteristics of ultra-early release (peak within 1-2 hours) and the indicator value of plaque instability, which shows certain potential in identifying myocardial injury associated with ACS.

### 4.2 Diagnostic evidence for heart-type fatty acid binding protein

Although H-FABP is often measured side-by-side with markers of oxidative stress such as MPO and d-ROMs, its biological origin and mechanistic basis are quite different: H-FABP is not involved in ROS generation or antioxidant defense pathways, but rather acts as a highly abundant, low-molecular-weight (15 kDa) fatty acid transporter in the cytoplasm of cardiomyocytes, which is released passively and rapidly after impairment of cell membrane integrity, such as ischemic injury. H-FABP mainly reflects the ischemia/myocardial injury axis, therefore, its inclusion in the multi-index detection panel can provide complementary information and reveal the pathophysiological process of AMI more comprehensively. The following clinical studies have evaluated its diagnostic performance in this context.

Recent advances in detection technology have begun to address the translational barriers for H-FABP. Novel platforms—including microfluidic chip-based automated systems and aggregation-induced luminescence nanoparticle biosensors—can quantify H-FABP within 15–25 minutes with detection limits in the low pg/mL to fg/mL range and excellent agreement with standard ELISA (39–41). Such improvements in analytical sensitivity and turnaround time are essential for incorporating H-FABP into point-of-care multi-marker panels. Nevertheless, these platforms have yet to undergo large-scale clinical validation, and assay standardization remains a prerequisite for their routine use in AMI diagnosis.

In terms of clinical research, H-FABP is prominent in the early identification of AMI. In the isolated heart perfusion model, the level of H-FABP was significantly correlated with the dynamic changes of cardiac function parameters such as left ventricular work (P < 0.05), suggesting that H-FABP can be used as a sensitive index of myocardial injury after ischemia, especially for the evaluation of donor heart function (42). However, its independent prognostic value remains controversial. A study of patients with suspected acute coronary syndromes but ultimately excluded AMI showed that H-FABP, as a non-necrotic marker, did not significantly improve long-term risk stratification, whereas high-sensitivity troponin T/I and GDF-15 showed greater predictive power (43). However, due to its rapid release kinetics and constantly optimized detection technology, H-FABP still has irreplaceable advantages in the diagnosis of AMI in the very early “time window”(especially within 3 hours after symptom onset), especially in the stage when high-sensitivity troponin has not yet increased significantly; H-FABP can be used as an important supplementary or auxiliary marker. The core advantage of H-FABP is that the sensitivity of H-FABP is significantly better than that of hs-cTn in the 1-3 H window period. Because of non-inflammatory/non-renal release, H-FABP has auxiliary differential value in ruling out non-ischemic cTn elevation.

## 5 The value of novel inflammatory oxidative stress markers for risk stratification of acute myocardial infarction

Recent studies have demonstrated the value of inflammation- and oxidative stress-related biomarkers in predicting short-term adverse outcomes after AMI (**Figure 3**). It must be emphasized that AUC values derived from different end points such as short-term mortality, severity of myocardial injury, or diagnostic accuracy in acute myocardial infarction cannot be directly compared. Interpretation must be based on the specific clinical problem, the characteristics of the population, and the performance of the test. The following are from non-AMI populations or experimental models In a cohort of 398 STEMI patients, a set of inflammatory markers at admission -including IL-6, IL-8, IL-10, FGF-21, FGF-23, ST1A1, MCP-1, 4E-BP1, and CST5 -were significantly associated with 28-day mortality (44). The predictive performance of this biomarker-based model was comparable to the GRACE score (AUC: 0.79 vs. 0.80), and combining the two further improved discrimination (AUC: 0.83, cNRI: 0.57, IDI: 0.08) (44). This finding underscores the additive value of inflammatory markers over clinical scores alone. Additionally, in 100 NSTEMI patients, a model incorporating MPO (an oxidative stress marker), IL-10, and hs-CRP yielded an AUC of 0.67 for identifying severe myocardial injury (defined by low LVEF and high TnI), suggesting moderate discriminative ability (14). Although this latter panel is not yet sufficient for clinical use, it illustrates the principle that simultaneous assessment of the inflammatory and oxidative stress axes may provide a more complete picture of post-infarction risk. Together, these studies support the concept that multi-marker panels rooted in the inflammation–oxidative stress dual-axis network hold promise for early risk stratification, though larger, multi-center studies with standardized assays are needed.

**Figure 3:**
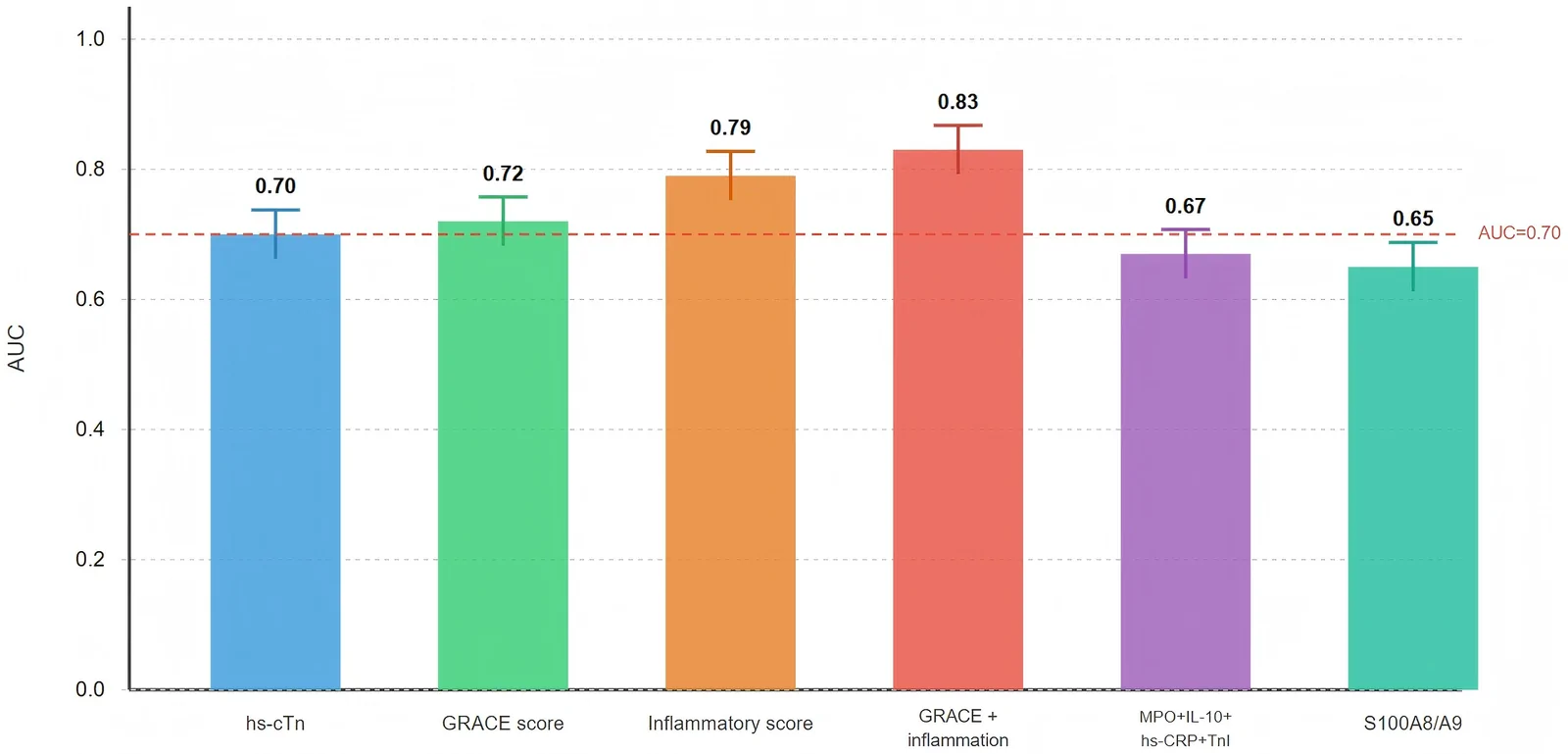
Comparison of area under the ROC curve (AUC) between single and combined models for predicting MACE after AMI.

Beyond short-term risk prediction, these inflammation- and oxidative stress-related biomarkers have also been linked to long-term cardiovascular outcomes, although direct evidence in post-AMI cohorts remains limited. In the general population-based PREVEND cohort, lower baseline levels of protein-adjusted free thiols—reflecting systemic oxidative stress—were independently associated with an increased risk of cardiovascular events (HR 0.63, 95% CI 0.40–0.99) and all-cause mortality (HR 0.52, 95% CI 0.32–0.85) in men (45). Similarly, elevated plasma d-ROMs (>400 UCARR), a global oxidative stress marker, predicted incident cardiovascular events and death independent of traditional risk factors (46). On the inflammatory axis, GDF-15 and IL-6 have been shown to independently predict composite cardiovascular endpoints, including myocardial infarction, in 3,817 patients with atrial fibrillation, with their integration significantly improving risk stratification accuracy (47). Although these studies were not conducted specifically in post-AMI populations, they suggest that markers aligned with the oxidative stress axis (free thiols, d-ROMs) and the inflammatory axis (GDF-15, IL-6) may jointly contribute to long-term risk assessment. Whether the dual-axis network concept can be extended to post-AMI long-term prognosis requires dedicated validation in AMI cohorts with serial biomarker measurements.

In the pathological process of acute myocardial infarction (AMI), inflammation and oxidative stress are not isolated parallel pathways, but closely intertwined and synergistically worsen myocardial injury through a multi-level, bidirectional amplification loop driven by positive feedback. The key biomarkers reviewed here - S100A8/A9 (inflammation axis core DAMP), MPO (oxidative stress/inflammation interface enzyme), H-FABP (early ischemic injury signal) and downstream effector molecules (e.g., IL-1β, ROS, MDA, Kyn, etc.) - do not simply coexist. However, a cascade network of “upstream triggering, midstream amplification, and downstream integration” runs through the whole process of myocardial injury.

In the upstream stage, mitochondrial dysfunction and reactive oxygen species (ROS) burst occur in the early stage of myocardial ischemia, leading to lipid peroxidation, protein oxidation and DNA damage. At the same time, necrotic cardiomyocytes rapidly release damage-associated molecular patterns (DAMPs) such as S100A8/A9 and HMGB1, activate innate immune receptors such as TLR4/RAGE, and initiate aseptic inflammation. Entering the midstream amplification stage, DAMPs and ROS form a positive feedback loop: S100A8/A9 further promotes ROS production and neutrophil extracellular traps (NETs) formation through the NF-κB/MAPK/PI3K-Akt pathway, while neutrophil-derived myeloperoxidase (MPO) catalyzes the production of HOCl, a potent oxidant. It can aggravate LDL oxidation, HDL dysfunction, endothelial injury and myocardial reperfusion injury, thus significantly amplifying the double harm of inflammation and oxidative stress. In the downstream integration stage, these processes jointly drive key clinical outcomes: they not only cause microvascular obstruction, myocardial stunning, and progressive myocardial necrosis, but also directly mediate adverse cardiac remodeling and heart failure, and are significantly associated with short-term major adverse cardiovascular events (e.g., MACE at 28 days), long-term cardiovascular events, and all-cause death. Therefore, the dynamic network of “trigger-amplification-integration” not only elucidates the pathological basis of S100A8/A9, MPO, H-FABP and other new biomarkers, but also provides a solid mechanism support for the construction of a multi-indicator diagnostic spectrum with inflammation and oxidative stress as the core.

## 6 Research progress of joint diagnostic models

High-sensitivity cardiac troponin (hs-cTn) is the cornerstone of AMI diagnosis but has well-recognized limitations: it may remain undetectable in the ultra-early phase after symptom onset, and its specificity is reduced in the presence of chronic myocardial injury, renal insufficiency, or other non-ischemic conditions (23). Crucially, hs-cTn signals myocardial necrosis, whereas the upstream pathological processes of inflammation and oxidative stress are not directly captured. This recognition has driven interest in combining hs-cTn with biomarkers that reflect the inflammatory and oxidative stress axes to achieve a multi-dimensional diagnostic and prognostic assessment. For instance, a panel incorporating MPO (oxidative stress), IL-10 and hs-CRP (inflammation), together with TnI, has been explored for identifying patients with severe myocardial injury (AUC 0.67) (14). While this specific combination showed only modest performance and focused on injury severity rather than early diagnosis, the concept of assembling markers from different pathogenic axes—the ischemic/necrotic, inflammatory, and oxidative stress—forms the foundation of the multi-marker diagnostic signature proposed in this review. Larger studies directly evaluating the incremental diagnostic yield of such dual-axis panels over hs-cTn in the early time window remain urgently needed.

While several multi-marker panels incorporating inflammation- and oxidative stress-related biomarkers have been proposed, none have been rigorously tested against a matched single-axis control panel in a prospective, cohort-internal design. Thus, the purported superiority of dual-axis integration—though strongly supported by pathophysiological rationale—remains a testable hypothesis awaiting validation.

The development of multi-omics and high-throughput detection platforms has paved the way for multi-marker diagnostic models in AMI. However, large-scale prospective studies that narrative evaluate the diagnostic performance of combining inflammation- and oxidative stress-related markers with hs-cTn are still lacking. Indirect evidence nonetheless supports the potential of such an approach. For instance, a scoring system incorporating inflammatory markers (IL-6, IL-8, MCP-1) significantly improved the prediction of 28-day mortality when added to the GRACE score (combined AUC 0.83 vs. GRACE alone), illustrating the incremental value of inflammatory axis information for risk stratification (44). Concurrently, emerging multiplexed detection platforms—such as electrochemical biosensor arrays and SERS-based nanosensors—have demonstrated the ability to simultaneously quantify multiple cardiac markers (e.g., CK-MB, cTnI, myoglobin) with ultra-high sensitivity (48). Although these platforms currently target conventional necrosis markers, they provide the technological foundation for integrating novel inflammatory and oxidative stress markers into a single panel. Critically, even the most advanced early diagnostic models, such as those evaluated in the APACE trial, have not yet incorporated inflammation- or oxidative stress-related biomarkers; the combination of cardiac myosin-binding protein C with hs-cTn achieved an AUC of 0.943 for NSTEMI, but markers like S100A8/A9, MPO, or GDF-15 were not included (49). This represents a clear opportunity: systematically embedding validated markers from the inflammatory axis and oxidative stress axis into existing high-sensitivity frameworks may further improve early diagnosis, particularly in the pre-troponin-rise phase (<1–1.5 h). Prospective trials that evaluate such dual-axis multi-marker panels are urgently warranted.

## 7 Limitations and challenges of the current study

Despite progress in identifying novel biomarkers of myocardial injury linked to inflammation and oxidative stress, their clinical translation faces significant hurdles, foremost among which is the substantial heterogeneity across studies. This heterogeneity manifests at multiple levels. First, study populations differ considerably in age, sex, comorbidities (e.g., renal insufficiency, metabolic syndrome), type of myocardial infarction (STEMI, NSTEMI, or MINOCA), and the presence of cardiogenic shock (44, 50). These variables can directly influence biomarker concentrations and their diagnostic performance—for instance, reduced renal clearance may elevate S100A8/A9 levels independently of myocardial injury, while the distinct pathophysiological profiles of STEMI and NSTEMI may alter the release kinetics of inflammatory versus oxidative stress markers. Such population-dependent variability not only complicates the establishment of universal reference ranges but also poses a challenge for constructing a broadly applicable multi-marker diagnostic signature within the inflammation–oxidative stress dual-axis framework.

Second, the lack of standardized sample collection time windows represents another major source of heterogeneity. Some studies obtain blood samples within the first hour of symptom onset, while others collect samples immediately or several hours after hospital admission (51). This variability is particularly problematic for inflammation- and oxidative stress-related biomarkers such as MPO and S100A8/A9, whose circulating levels change rapidly within the first few hours after myocardial injury. Without a uniform sampling protocol, comparisons of marker kinetics across studies become unreliable. In addition, differences in detection platforms and methodologies further compromise the comparability of results. Biomarker measurement currently employs a wide range of techniques, from discovery-phase high-throughput platforms (e.g., OLINK, DIA proteomics) to clinically oriented biosensors and microfluidic chips, yet the analytical sensitivity, specificity, linear range, and inter-assay reproducibility of these platforms remain unstandardized (3, 52–55). This methodological fragmentation makes it difficult to establish consistent cutoff values for inflammatory- and oxidative stress-axis markers, thereby impeding their integration into a unified multi-marker diagnostic signature. Together with population heterogeneity, these technical sources of variation weaken the reproducibility and generalizability of current evidence and delay the clinical translation of novel dual-axis biomarkers.

Beyond cohort heterogeneity and assay platform differences, pre-analytical factors represent a major yet often underappreciated source of analytical variability—particularly for oxidation- and inflammation-related biomarkers. First, sample matrix matters: S100A8/A9 and HMGB1 show higher stability in EDTA plasma than in serum, likely due to reduced ex vivo release from activated leukocytes during clot formation; conversely, MPO activity is best preserved in citrate plasma with immediate protease inhibition, as serum clots may trigger neutrophil degranulation and artifactual elevation. Second, storage conditions critically impact oxidative stress markers: malondialdehyde (MDA) and d-ROMs are highly susceptible to auto-oxidation at room temperature or after repeated freeze-thaw cycles; studies report up to 30% degradation of free thiols after one freeze-thaw, necessitating strict –80 °C storage and single-use aliquoting. Third, circadian rhythm may confound interpretation: circulating IL-6 and MPO exhibit diurnal variation (peak ~04:00–06:00), potentially affecting absolute concentrations independent of AMI status. These pre-analytical variables—not only compromise intra- and inter-laboratory reproducibility but also undermine the validity of pooled analyses and meta-research. Therefore, future multicenter validation studies must adopt harmonized pre-analytical protocols aligned with the Clinical and Laboratory Standards Institute (CLSI) C62-A guidelines for oxidative stress biomarkers and the International Society on Thrombosis and Hemostasis (ISTH) recommendations for DAMPs.

Furthermore, although multi-marker panels hold considerable promise, technical complexity and high cost constitute a major practical barrier to clinical implementation. For instance, microfluidic chip-based platforms capable of simultaneous six-marker detection can overcome spectral overlap and achieve unbiased multiplexed analysis; however, such systems remain expensive and require specialized operator training, limiting their deployment in primary care settings (55). Importantly, most of these advanced platforms have been validated only for conventional cardiac markers, and their extension to the simultaneous detection of inflammation-axis (e.g., S100A8/A9) and oxidative stress-axis (e.g., MPO) markers has yet to be demonstrated. Coupled with the lack of standardized detection protocols, unified reference intervals, and robust cost-effectiveness data, these technical and logistical obstacles continue to widen the research-to-clinic translation gap. Without addressing these issues, the vision of a dual-axis multi-marker diagnostic signature for early AMI diagnosis and risk stratification will remain unrealized.

Despite the encouraging progress, the clinical translation of inflammation- and oxidative stress-related biomarkers for AMI remains challenging. Future research should prioritize several key directions. First, large-scale, multicenter, prospective cohort studies are needed to validate the diagnostic robustness and cross-population generalizability of the candidate markers discussed in this review—particularly S100A8/A9 (inflammatory axis), MPO (oxidative stress/inflammatory interface), H-FABP (ischemic axis), and GDF-15 (inflammatory/prognostic axis)—within a unified multi-marker panel. Second, standardization and the development of point-of-care technologies must be extended beyond conventional necrosis markers. Current multiplexed POCT platforms predominantly target cTnI, CK-MB, and myoglobin; future efforts should incorporate inflammatory and oxidative stress markers to realize true dual-axis multi-marker detection at the bedside. Third, deeper mechanistic and causal investigations are essential. Mendelian randomization studies have suggested a causal role for S100A8/A9 in heart failure, while damage profiling of telomeric DNA has demonstrated that oxidative stress and inflammatory stress leave distinct molecular signatures at the nucleotide level. Such mechanistic insights not only provide a biological rationale for the dual-axis network concept but also guide the selection of more specific, pathology-driven biomarkers and inform targeted therapeutic strategies. As a narrative review, our synthesis reflects expert interpretation rather than quantitative pooling of effect sizes, and thus does not replace future systematic reviews or meta-analyses. Collectively, these efforts will be required to bridge the gap between exploratory research and the clinical implementation of a dual-axis multi-marker diagnostic signature for AMI.

## 8 Conclusions

Early and accurate diagnosis of AMI remains challenging. While hs-cTn has improved sensitivity, it is limited by the troponin-blind period and reduced specificity in non-ischemic conditions. Inflammation and oxidative stress, as intertwined pathogenic mechanisms, have driven the discovery of novel biomarkers. Viewed through the inflammation–oxidative stress dual-axis network, S100A8/A9 and IL-6 reflect the inflammatory axis, MPO and d-ROMs the oxidative stress axis, and H-FABP the early ischemic axis. Current evidence supports combining these axis-specific markers into a multi-biomarker diagnostic signature to complement hs-cTn for early diagnosis and risk stratification. However, existing data are constrained by small cohorts, population heterogeneity, inconsistent sampling windows, and unstandardized assays. Future priorities include large-scale multicenter validation of dual-axis panels, development of point-of-care platforms capable of simultaneous detection of inflammatory and oxidative stress markers, and rigorous cost-effectiveness evaluation. Advancing from a single-marker, necrosis-centered strategy toward a dual-axis, multi-dimensional framework may ultimately enable earlier diagnosis, more precise risk assessment, and individualized management of AMI.

Importantly, the conceptual framework of a ‘dual-axis’ signature reflects a mechanistically grounded hypothesis—not an established clinical reality. To date, no study has directly compared the diagnostic or prognostic yield of a dual-axis panel versus rigorously matched single-axis alternatives within the same AMI cohort under standardized conditions. Future validation must therefore prioritize head-to-head, hypothesis-driven trials with pre-specified non-inferiority/superiority endpoints, rather than merely reporting incremental AUC improvements over conventional benchmarks.
